# Red Blood Cell Distribution Width Is a Biomarker of Red Cell Dysfunction Associated with Inflammation, Macrophage Polarization, and Coronary Artery Calcification: A Prognostic Marker in Cardiovascular Disease and a Potential Target for Anti-Cytokine Therapy

**DOI:** 10.3390/life16091512

**Published:** 2026-09-10

**Authors:** Kitty Inzunza-Esparza, Artemio García-Escobar

**Affiliations:** 1 Institute for Development and People, Francisco de Vitoria University, M-515, km 1, 800, 28223 Madrid, Spain; 2 Pontificio Instituto Teológico Juan Pablo II Madrid, UCAM, Pl. del Conde de Barajas, 1, Centro, 28005 Madrid, Spain; 3 Cardiology Department, Quironsalud University Hospital Madrid, Calle Diego de Velázquez, 1, 28223 Madrid, Spain; dr_garciaescobar@hotmail.com; 4 Cardiology Department, Ruber Juan Bravo Quironsalud University Hospital, Calle de Juan Bravo, 49, 28006 Madrid, Spain

**Keywords:** red blood cell distribution width, inflammation, interleukin-6, macrophage polarization, coronary artery calcification, hepcidin, ferritin, atherosclerotic cardiovascular disease, anti-cytokine therapy, clonal hematopoiesis of indeterminate potential

## Abstract

Coronary heart disease represents a great economic expense, especially coronary artery calcification (CAC), which is associated with worse clinical outcomes and increased technical complexity of percutaneous coronary intervention. Red cell distribution width (RDW) is a marker of dysregulated erythropoiesis associated with inflammation and iron accumulation in macrophages that is mediated by hepcidin. Research has indicated that higher RDW levels are linked to an increased risk of adverse cardiovascular events, and some studies have shown an association with CAC. This narrative review aims to summarize the current evidence on the mechanisms responsible for elevated RDW and its relationship with inflammation and CAC. Systemic inflammation induces the expression of hepcidin, which alters iron levels; macrophages, depending on iron levels, can help to resolve inflammation or trigger inflammation in atherosclerotic cardiovascular disease (ASCVD) and can induce vascular calcification. This is known as macrophage polarization. Evidence from large-scale clinical trials has established the involvement of immune-mediated inflammation in the pathogenesis of ASCVD. Thus, systemic inflammation is a novel cardiovascular risk factor that can be treated with anti-cytokine therapy. RDW could be a useful marker of inflammation associated with CAC and a risk marker for experiencing adverse cardiovascular events.

## 1. Complexity and Economic Impact of Coronary Artery Calcification

At the turn of the 21st century, coronary heart disease (CHD) affected approximately 8% of the US population, and its incidence was projected to increase progressively [1]. A statistical analysis from the National Health and Nutrition Examination Survey (NHANES) reported that by 2060, compared with 2025, the number of people with CHD will increase by 31.1% (21.9 million to 28.7 million). In addition, the study predicts that future increases in cardiovascular risk factors such as diabetes mellitus (DM) will increase by 39.3% (39.2 million to 54.6 million), hypertension by 27.2% (127.8 million to 162.5 million), and dyslipidemia by 27.5% (98.6 million to125.7 million) [2]. Moreover, at the beginning of this century, there were an estimated 5.8 million new cases of CHD in the 57 European Society of Cardiology member countries in Europe [3]. Furthermore, an observational study in China reported that the crude prevalence of CHD was 4.42% [4]. In the last two decades, in the U.S., there has been an increase in percutaneous coronary intervention (PCI) since the introduction of the drug-eluting stent (DES), whereas coronary artery bypass graft surgery has been decreasing [5]. In addition, the advances in PCI techniques and the increase in the octogenarian population have led to the position of PCI as the most frequent type of revascularization in routine clinical practice.

Vascular calcification is associated with traditional atherogenic risk factors such as advanced age, DM, hypertension, dyslipidemia, and chronic kidney disease (CKD) [6]. In current registries and meta-analyses, moderate and severe coronary artery calcification (CAC) prevalence ranges between 18% and 32% [7,8,9]. CAC is associated with worse clinical outcomes, partly related to comorbidity and the increased technical complexity of PCI that can affect the crossing of the lesion and optimal stent expansion, leading to higher rates of intraprocedural complications, such as no-reflow, coronary dissection, or perforation. Furthermore, CAC is related to increased major cardiovascular adverse events (MACE) such as definite stent thrombosis, myocardial infarction (MI), ischemic target lesion revascularization, target vessel revascularization, and death [8,9]. In this regard, the current guidelines recommend the use of plaque preparation techniques to treat CAC such as rotational atherectomy (RA), excimer laser coronary atherectomy (ELCA), orbital atherectomy or intravascular lithotripsy (IVL) [10]. The latter is a new method used to treat calcified coronary lesions; some studies suggest that IVL has fewer intraprocedural complications than RA [11]. Recently, IVL has proven its effectiveness in different calcium morphologies (concentric and eccentric calcification) [12]. Moreover, in situations of balloon-uncrossable and the combination of IVL with different plaque preparation techniques such as RA has been reported with good results [13], as well as in the case of microcatheter-uncrossable lesions, the combination of ELCA with IVL (ELCATripsy) has also been reported with successful results [14]. Despite all these measures, in the first decade of this century, an estimated $108.9 billion was spent annually on CHD treatment [15]. In addition, a recent 10-year health care cost analysis revealed the CAC score ≥ 400 as one of the main variables identifying a high-risk population cohort that consumed nearly one-half of the estimated $155 million for cardiovascular disease [16]. Therefore, the CAC poses a clinical challenge in managing CHD with important economic impact. For this reason, finding novel prognostic markers for cardiovascular disease that are associated with CAC could improve the cardiovascular risk assessment and identify the subset of patients with a greater likelihood of moderate-severe CAC that will require the use of plaque preparation techniques to treat CAC.

## 2. Red Blood Cell Distribution Width and Its Association with Inflammation, Macrophage Iron Retention, and Coronary Artery Calcification: Background, Methods and Results

Many cohort studies have reported the relationship between red blood cell distribution width (RDW) and CAC [17,18,19,20,21,22,23,24]. Over the last two decades, molecular medicine has turned toward a comprehensive understanding of the dynamic processes of vascular calcification. On this point, a serial in vivo study and positron-emission tomography in humans have corroborated that inflammation is an important factor that precedes and promotes vascular calcification [25,26]. Moreover, preclinical studies have proven that macrophages are involved in the pathogenesis of CAC [27]. Similarly, there has been more than enough research that has proven the macrophages’ role in the pathogenesis of ASCVD [28,29,30,31]. In fact, the presence of macrophages seen by intracoronary imaging in an atherosclerotic plaque is associated with plaque calcification [32,33,34], is also a predictor of rapid progression of CAC [35], and is associated with increased risk of MI and cardiovascular death [36]. Systemic inflammation induces the expression of hepcidin that alters iron levels; macrophages, depending on iron levels, can initiate inflammation or help resolve it [27].

This narrative review aims to summarize the current evidence on the mechanisms responsible for elevated RDW and its relationship with inflammation and CAC. We conducted a narrative review using a structured literature search on PubMed with the terms “red blood cell distribution width and calcification”; only studies that mentioned coronary arteries were included, with no time frame restriction, and the language restriction was to English. For additional studies, reference lists of selected articles were manually reviewed using a snowballing approach. Of note, all data curation and discrepancies were resolved through discussion and consensus. In addition, we highlighted the most relevant studies regarding the stimulus and conditions that can activate the macrophages toward an inflammatory response in the context of CHD and CAC.

A total of 8 cohort studies were included (Table 1), comprising 16,003 subjects. RDW-CV cut-off values associated with CAC ranged from 13.05% to 14.4%, while RDW-SD values ranged from 43.7 to 46.8 fL. The reported thresholds varied according to the study population, hematological analyzer, and definition of calcification. Of note, one study found that higher RDW-SD was associated with severe coronary calcification (CACS > 400) [18], while another study demonstrated an independent association between increasing RDW-SD and coronary calcification after adjustment for several cardiovascular risk factors [17]. Moreover, other research revealed that high RDW-CV independently predicted adverse cardiovascular events [20]. Hence, high RDW-SD is associated with a greater burden of calcification, whereas RDW-CV is associated with an increased risk of suffering adverse cardiovascular events.

## 3. Hepcidin Molecular Structure and Functions

Hepcidin is a tightly folded 25-residue peptide hormone synthesized by the liver and was discovered two decades ago. Hepcidin hepatic production is stimulated by iron loading and inflammation. Hepcidin inhibits iron transport by binding to the iron export channel ferroportin, which is located in intestinal epithelial cells, reticuloendothelial cells (macrophages), hepatocytes, and erythrocytes. In this context, hepcidin is a key regulator of iron homeostasis in many pathological states and plays an important role in regulating erythropoiesis and erythrocyte turnover [37]. Therefore, hepcidin mRNA production is reduced by hypoxemia due to low oxygen concentration or anemia caused by blood loss or hemolysis. Conversely, hepcidin mRNA production rises with inflammation and in response to iron overload [37].

CKD reduces renal excretion of hepcidin, which increases plasma levels [38]. The clearance of hepcidin via hemodialysis suggests a therapeutic intervention in which patients with CKD, anemia, and high hepcidin levels are changed to a more intensive dialysis regimen that could improve iron utilization [39].

In contrast, evidence supports the fact that the interleukin (IL)-6 stimulates the hepcidin production through direct binding of signal transducer and activator of transcription 3 (STAT3) [40]. IL-6 is Interleukin-6 (IL-6) is a well-characterized cytokine that plays a crucial role in the inflammatory response associated with conditions such as infectious diseases [41], oncological diseases [41], immune-related disorders [41], DM [42,43], heart failure (HF) [44], and cardiovascular disease [45,46].

## 4. The Role of Macrophages in Atherosclerotic Cardiovascular Disease

In recent years, large-scale clinical trials have provided compelling evidence that immune-mediated inflammatory pathways play a critical role in atherosclerotic cardiovascular disease (ASCVD). Both the innate immune system, including neutrophils and macrophages, and the adaptive immune system, particularly T cells and other lymphocyte populations, contribute to the development and progression of atherosclerosis and atherothrombosis [47].

An atherosclerotic plaque morphology analysis showed that smooth muscle cells dominate in the fibrous cap, which also contains many T cells; the lipid core is dominated by macrophages [48]. Thus, macrophages play a central role in the development of ASCVD, and plasticity is a hallmark of cells of the monocyte–macrophage lineage in response to local microenvironmental stimuli [49]. Within the context of ASCVD, macrophages are broadly categorized into two phenotypic subsets. Classically activated M1 macrophages are primarily associated with the initiation and perpetuation of inflammatory responses, whereas alternatively activated M2 macrophages are generally involved in the resolution of inflammation and the restoration of tissue homeostasis [49]. In vitro, M1 macrophage polarization can be induced by a range of proinflammatory stimuli, including Toll-like receptor (TLR) ligands, interferons, pathogen-associated molecular patterns (PAMPs), lipopolysaccharide (LPS), and atherogenic lipoproteins [50,51,52]. M1 macrophages contribute to tissue destruction and secrete proinflammatory factors, including high levels of IL-1β, IL-6, and tumor necrosis factor-alpha (TNF-α) [52,53]. In accordance with their proinflammatory phenotype, M1 macrophages display enhanced activation of pivotal inflammatory transcriptional programs, notably those governed by nuclear factor-κB (NF-κB) and signal transducer and activator of transcription (STAT) signaling [54]. M2 macrophages are polarized in response to a combination of the cytokines IL-4, IL-10, and TGFβ, as well as IL-4 and IL-13 and secrete anti-inflammatory factors such as the IL-1 receptor agonist and collagen [55,56,57]. M2 macrophages are characterized by their expression of CD163, mannose receptor 1, resistin-like molecule, and high levels of arginase-1 [58,59,60]. Overall, the conventional M1/M2 dichotomy represents an overly simplistic framework that fails to fully capture the considerable phenotypic and functional heterogeneity of macrophages. Nevertheless, M1 macrophages are classically induced by stimuli such as lipopolysaccharide (LPS), are preferentially enriched within actively progressing atherosclerotic plaques, and exhibit elevated expression of inducible nitric oxide synthase (iNOS), resulting in increased nitric oxide (NO) production [61,62,63], whereas M2 macrophages express high levels of arginase, promote plaque stability, and contribute to tissue repair [60,64].

## 5. The Iron Homeostasis Role in the Macrophage Polarization

Basic science studies demonstrated that oxidized low-density lipoproteins (LDL) do not induce polarization towards M1 or M2 macrophage phenotypes. Thus, the polarization of macrophages is determined by other microenvironmental factors [65].

**Figure 1:**
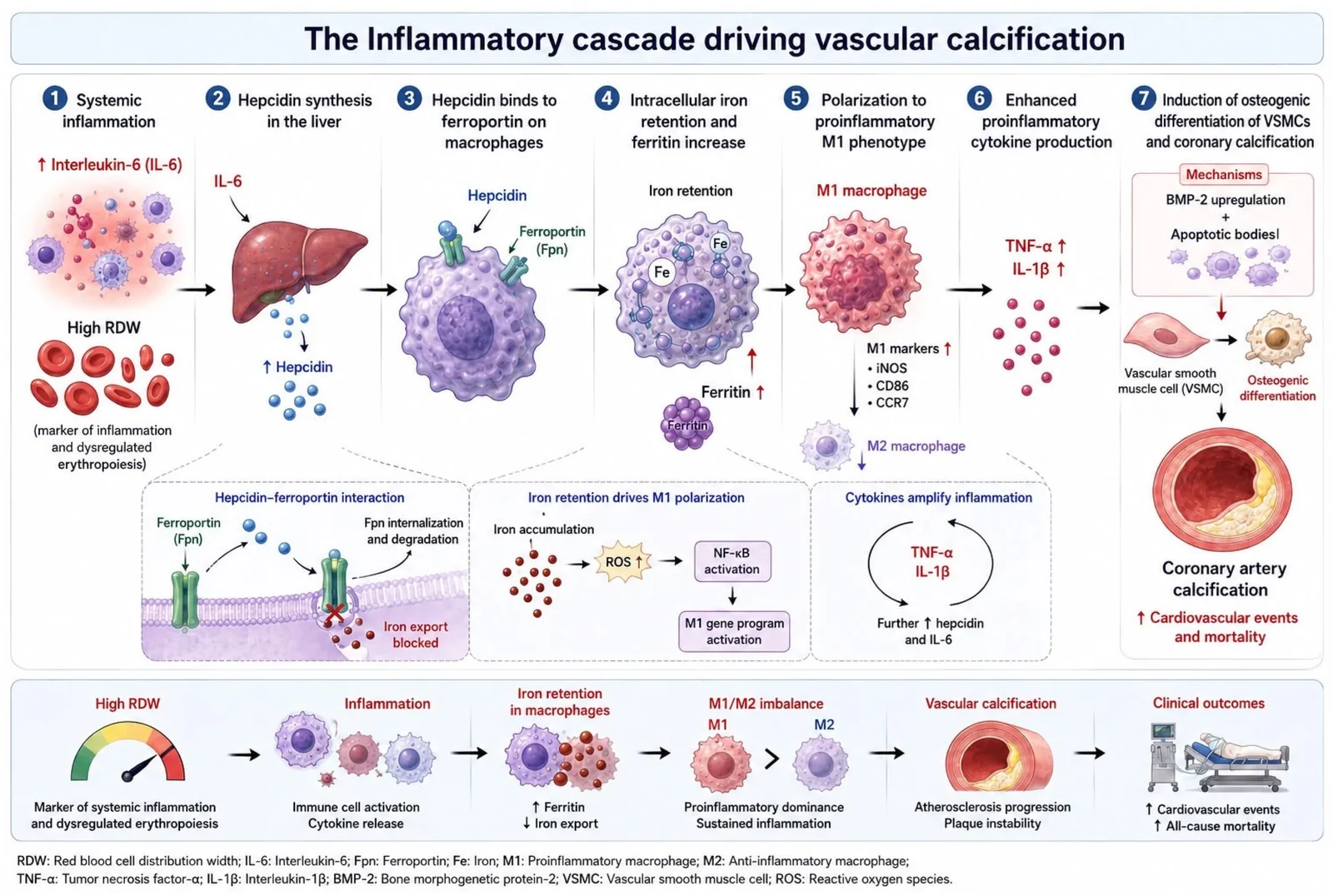
The inflammatory cascade driving vascular calcification. The blue arrow ↑ means the increasing blood levels of hepcidin. The red arrow ↑ means the increasing levels of in-tracellular ferritin (macrophage). The black arrow ↑↓ means that the macrophage accumulates ferritin and decreases iron release from macrophages.

In this regard, preclinical studies showed that low intracellular iron levels in macrophages inhibit the expression of proinflammatory cytokines [66,67], whereas high intracellular iron levels induce TNF-α and IL-6 [68,69]. Interestingly, the addition of Fe^2+^ increased TNF-α release in Kupffer cells [69]. Similarly, in vitro studies found that reduced macrophage iron content was associated with decreased expression of M1 phenotypic markers [70,71], whereas iron overloading of macrophages induced in situ an unrestrained proinflammatory M1 activation state [72]. Furthermore, treatment of human macrophages in vitro with the iron chelator deferiprone decreased LPS-induced NO synthase expression and reduced the uptake of oxidized LDL relative to iron-treated cells [73]. These results highlight the importance of reducing intracellular macrophage iron, which has an anti-inflammatory and atheroprotective effect [70,71,73]. Moreover, intraplaque hemorrhage (IPH) is considered a major factor for plaque instability and accelerated progression [74]. An autopsy study found that calcifications were strongly associated with the presence of IPH [75]. In addition, An in vivo analysis showed that IPH with lysed erythrocyte membranes, but not intact erythrocytes, promoted the osteoblastic differentiation of smooth muscle cells via BMP-2 and apoptotic bodies that led to vascular lesion calcification [76]. A preclinical study demonstrated that IPH can alter macrophage iron content in atherosclerotic lesions, and also showed that decreasing intracellular iron in macrophages provokes a reduction in reactive oxygen species production, which increases the macrophage ATP-binding cassette transporter expression and cholesterol efflux to apolipoprotein A-I [77]. In summary, the inflammatory cascade driving vascular calcification begins with elevated interleukin-6 (IL-6), reflecting systemic inflammation, which stimulates hepatic hepcidin synthesis. Increased hepcidin levels promote binding to ferroportin on macrophages, thereby inhibiting iron export and leading to intracellular iron retention and increased ferritin levels. This iron accumulation may, in turn, promote the polarization of macrophages toward a proinflammatory M1 phenotype, with enhanced production of proinflammatory cytokines such as TNF-α and IL-1β. These inflammatory processes may contribute to the osteogenic differentiation of vascular smooth muscle cells through mechanisms involving bone morphogenetic protein-2 (BMP-2) and the release of apoptotic bodies, ultimately promoting the development and progression of coronary artery calcification (Figure 1). The role of RDW in this chain: iron imbalance and an inflammatory cytokine background lead to ineffective erythropoiesis and the release of reticulocytes of varying sizes, which is reflected in an increase in RDW; however, RDW itself is not an active participant in the pathogenesis, but rather an integral marker of these disorders.

## 6. Ferritin and Its Association with Insulin Resistance

Another cohort study of NHANES revealed that higher ferritin and lower transferrin saturation are associated with a higher risk of preDM in the general population [78]. Increased ferritin concentrations are associated with markers of early CHD, independently of traditional cardiovascular risk factors, including the Framingham risk score [79].

Excess iron, once stored in the liver, interferes with glucose metabolism, causing hyperinsulinemia via both decreased insulin extraction and impaired insulin signaling. Moreover, insulin enhances the uptake of extracellular iron, inducing the redistribution of transferrin receptor 1 to the cell surface while downregulating hepcidin expression [80].

The registry of the Verona Heart Project reported higher ferritin levels in subjects with metabolic syndrome (MetS) than in control; the presence of C282Y heterozygosity was not significantly different between MetS subjects with or without hyperferritinemia [81]. A cross-sectional study of 6044 participants in the third NHANES found that, after excluding individuals with hemochromatosis, elevated serum ferritin values were positively associated with the prevalence of the MetS and with insulin resistance [82]. Acquired abnormalities of iron metabolism range from dysmetabolic hyperferritinemia without intrahepatic iron deposition to hyperferritinemia with mild to moderate liver iron concentration, also called dysmetabolic-hepatic iron overload syndrome [80]. In addition, a prospective study of noncirrhotic patients with hepatitis C virus (HCV) infection found that, DM but not HCV infection, was independently associated with serum ferritin concentrations. These findings suggest that DM, rather than HCV infection per se, is the principal determinant of the elevated ferritin levels observed in patients with chronic HCV infection [83]. In contrast, DM is associated with early age-related macular degeneration (AMD) [84]. A study demonstrated higher iron levels in the eyes of patients with AMD than in the eyes of age-matched controls. In a preclinical model, iron chelation with ferriprox ameliorated oxidative stress and protected against iron overload-induced retinal degeneration [85]. As mentioned earlier, the treatment of human macrophages in vitro with an iron chelator reduced the uptake of oxidized LDL relative to iron-treated cells [73]. Moreover, the Trial to Assess Chelation Therapy (TACT) study included patients aged ≥ 50 with prior MI who received systemic chelation therapy with an intravenous weekly low dose (3 g) of ethylenediaminetetraacetic acid (EDTA). Those with DM had a marked reduction in the risk of the composite outcome of cardiovascular mortality, stroke, or MI without significant adverse effects related to the EDTA. The sub-analysis revealed that diabetic patients on insulin therapy at baseline may accrue the greatest benefit of chelation therapy [86]. Nevertheless, more research is required to find out if this systemic chelation therapy regimen can produce a sustained decrease in intracellular iron levels in macrophages, and further clinical trials are needed to confirm whether systemic chelation therapy reduces MACE in DM.

## 7. Red Blood Cell Distribution Width Definition

**Figure 2:**
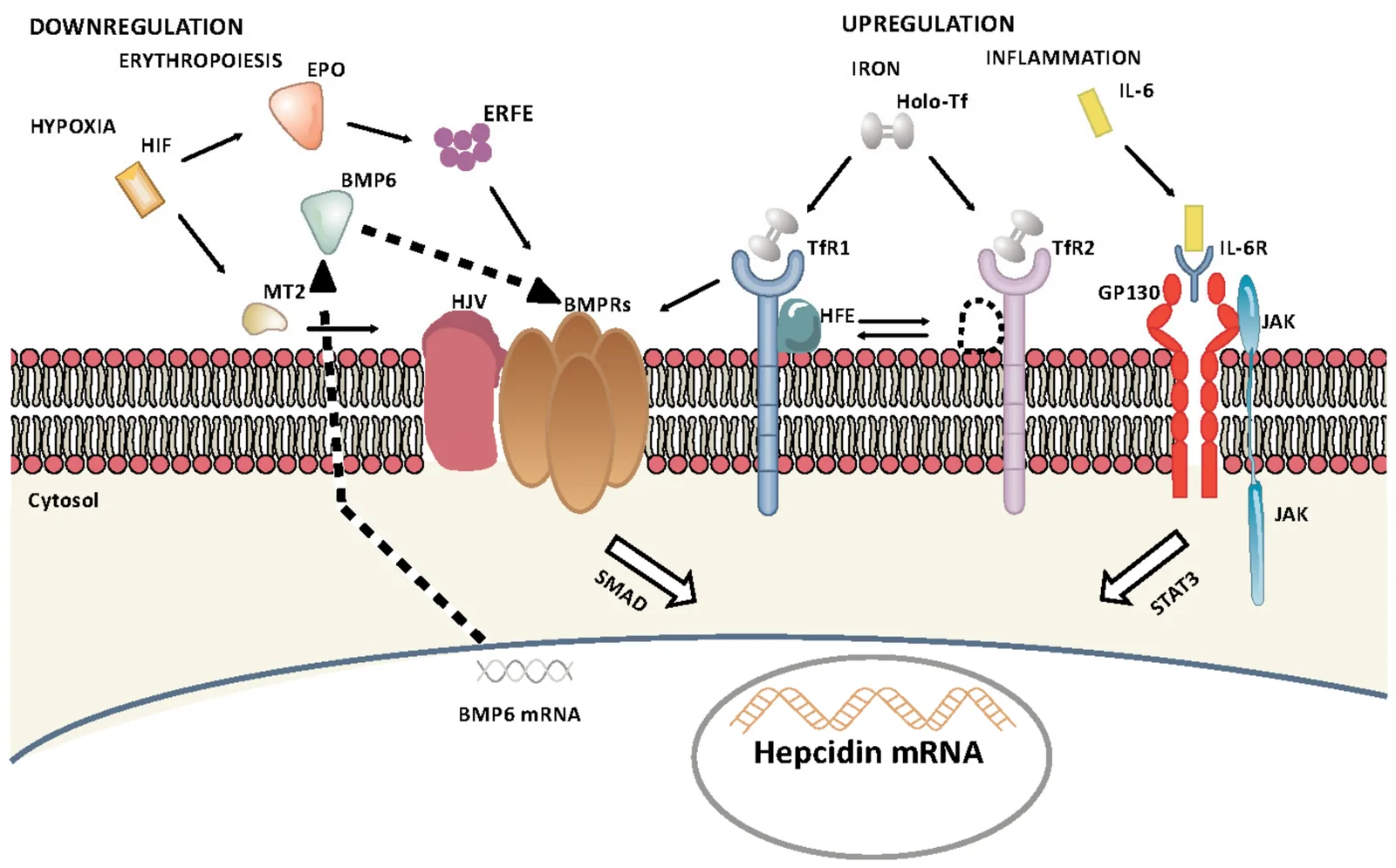
Regulation of hepcidin expression. Hepcidin synthesis is tightly regulated by systemic iron availability, inflammation, hypoxia, and erythropoietic activity. Iron regulation: An increase in serum transferrin saturation promotes the displacement of HFE from transferrin receptor 1 (TfR1), allowing HFE to interact with transferrin receptor 2 (TfR2) and form the HFE/TfR2 complex. This complex promotes hepcidin transcription through activation of the hemojuvelin (HJV)/bone morphogenetic protein (BMP)/SMAD signaling pathway [90]. In hepatocytes, increased intracellular iron also stimulates BMP-6 expression. Secreted BMP-6 subsequently interacts with HJV and activates BMP/SMAD signaling, thereby enhancing hepcidin production. Inflammatory regulation: Interleukin-6 (IL-6) binds to the IL-6 receptor α/gp130 complex, triggering Janus kinase (JAK)-signal transducer and activator of transcription 3 (STAT3) signaling and consequently inducing hepcidin transcription [40]. Hypoxic regulation: Hypoxia-inducible factors (HIFs) suppress hepcidin expression through at least two mechanisms: stimulation of erythropoietin (EPO) production and induction of transmembrane serine protease 6 (TMPRSS6), which encodes matriptase-2 (MT2). Matriptase-2 promotes the shedding of membrane-bound HJV and attenuates the responsiveness of the hepcidin promoter to BMP-mediated signaling [91]. Erythropoietic regulation: EPO stimulates the production of the erythroid-derived regulator erythroferrone (ERFE). ERFE suppresses hepcidin synthesis by binding and sequestering BMP ligands, thereby limiting BMP/SMAD pathway activation and facilitating iron availability for erythropoiesis [92].

RDW is a parameter included in a routine full blood count that measures how varied the red blood cells (RBC) are in size and volume, which is performed by automated cell analyzers as a standard part of any hematology analysis [87]. Automated blood cell analyzers typically report RDW in two formats, the first as RDW-CV (Coefficient of variation), which is the most common format, and the second as RDW-SD (Standard deviation) [17,87,88]. The RDW-CV is calculated from the coefficient of variation in the RBC size distribution histogram. Thus, this is calculated as RDW = standard deviation of RBC volume × 100 ÷ mean corpuscular volume (MCV) with reference values of approximately 11–15% [87,88]. The RDW-SD is a direct measurement of the histogram width at the 20% height level; the result is reported in femtoliters (fL) and is not influenced by the MCV, with reference values of 39 to 46 fL [17]. Therefore, a high RDW reflects a more heterogeneous population (large variation) in RBC size, a morphological feature referred to as anisocytosis, whereas a lower RDW reflects a relatively homogeneous population (low variation) of RBC sizes. Given the central role of hepcidin in maintaining systemic iron homeostasis and the essential contribution of iron availability to erythropoiesis, dysregulation of hepcidin expression, whether through suppression or upregulation, may alter erythrocyte production and maturation [89], thereby increasing variability in red blood cell size and consequently elevating RDW (Figure 2).

## 8. RDW as a Biomarker of Red Cell Dysfunction and Its Association with Inflammation

RDW is a parameter included in routine full blood count. It is feasible, quick, and easy to obtain at the bedside. A high RDW can be due to ineffective erythropoiesis, inflammation, hypoxemia, and/or impaired iron availability [87,88,89]. Hence, RDW is not a specific marker of cardiovascular disease since high RDW can be found in conditions of impaired haematopoiesis, such as nutritional deficiencies (iron, folate, vitamin B12), hematologic disorders such as some haemoglobinopathies, myelodysplastic syndrome, myelophthisic anemia, CKD (low eritroproetin production), also in conditions of high systemic inflammation such as autoimmune disorders, oncological disease, critical illness processes such as acute respiratory distress syndrome, and finally in conditions of increased red cell destruction, such as hemolysis and acute bleeding, or when different populations of RBC are present, such as after blood transfusion [87,88]. According to the literature evidence, many studies have proven that a high RDW is associated with an increased risk of all-cause mortality and adverse cardiovascular events [87,88,89]. From a practical perspective, a high RDW is associated with inflammation with high levels of cytokines such as IL-6 [93] that stimulate the hepcidin, which leads to macrophage iron retention (increased ferritin levels), higher ferritin levels produce insulin resistance [82] and increase the polarization of the macrophages from M2 (inducing inflammation resolution) to M1 (triggering inflammation) in ASCVD which can induce vascular calcification [68,69,70,73,76,77]. Of note, the CARDIA (Coronary Artery Risk Development in Young Adults) study reported greater CAC in diabetes with long-term high insulin resistance [94], and DM is associated with high IL-6 levels [95]. Similarly, the CRIC (Chronic renal insufficiency cohort) study demonstrated a graded relationship between lower estimated glomerular filtration rate and increasing CAC independent of traditional risk factors [96]. In this regard, CKD impairs hepcidin clearance, leading to its accumulation in plasma [38,39]. DM and CKD are associated with high RDW [97,98,99] and are the most common cardiovascular risk factors associated with coronary calcification [5,82,94,96]. In addition, aging is associated with increased levels of hepcidin [100] and with coronary calcification [101].

Many studies and meta-analyses have proved that clonal hematopoiesis of indeterminate potential (CHIP) is associated with an increased risk of ASCVD and all-cause mortality as well as with CAC [102,103,104]. CHIP is defined as the presence of hematopoietic stem cells or other early blood cell progenitors (blood-cell clones) that contribute to the formation of a genetically distinct subpopulation of blood cells in persons without other hematologic abnormalities [103]. Clonal hematopoiesis usually arises from somatic mutations in hematopoietic stem and progenitor cells in genes such as TET2, ASXL1, DNMT3A, or JAK2, which lead to clonal expansion of hematopoietic cells. The prevalence of clonal hematopoiesis increases with age and confers an increased risk of hematological malignancies [104,105]. However, some clonal hematopoiesis cases could arise in the absence of somatic gene mutations as a result of neutral drift acting on a small population of active hematopoietic stem cells, which is common in the elderly [106]. Prospective cohort studies reported that CHIP is associated with CAC and CHD [103]. Among the common genetic variants that give rise to clonal hematopoiesis, those which increase JAK-STAT signaling had a higher risk for CHD (12.1-fold risk increase) compared to other genetic variants such as DNMT3A, TET2, and ASXL1 (1.7 to 2.0 fold risk increase) [103]. Of note, nearly all individuals with CHIP do not exhibit abnormal blood cell counts or other symptoms of hematologic disease [107]. In fact, the only significant difference in blood-cell indexes was a significant increase in RDW and this difference was driven by a large increase in this variable in a subgroup of persons with mutations [103]. These studies suggest that CHIP-associated genes linked to CHD may increase the risk of coronary events due to altered transcriptional output of macrophages which mediates the expression of proinflammatory cytokines.

## 9. High Systemic Inflammation in Atherosclerotic Cardiovascular Disease: A Target for Anti-Cytokine Therapy

Given that the considerable evidence demonstrated the operation of the immune system and inflammatory pathways in the development of ASCVD, some clinical trials showed that the use of anti-inflammatory therapy targeting the immune system by modulating or blocking interleukins, also known as anti-cytokine therapy, reduced the risk of cardiovascular events in patients with established ASCVD [47]. Therefore, high systemic inflammation emerges as a novel cardiovascular risk factor and target for anti-cytokine therapy. In this context, in June 2023, the U.S. Food and Drug Administration (FDA) approved once-daily colchicine at a dose of 0.5 mg for the reduction of cardiovascular events in patients with established ASCVD who exhibit persistent systemic inflammation, defined by a C-reactive protein (CRP) concentration of ≥2 mg/L [108].

Colchicine is an old anti-inflammatory drug derived from the plant Colchicum autumnale [109]. Although the exact mechanism of action is not fully understood, according to the current evidence, colchicine was found to inhibit the NLRP3 (nucleotide-binding oligomerization domain-, leucine-rich repeat-, and pyrin domain-containing protein 3) inflammasome, also known as NALP3, which mediates crystal-induced inflammation present in gout attributable to uric acid crystals and in atherosclerosis attributable to cholesterol crystals [110,111,112]. NLRP3 inflammasomes are present in the cytoplasm of neutrophils and macrophages [109,112]. In this regard, colchicine disrupts the microtubule structure and blocks the assembly of the NLRP3 inflammasome. This results in reduced secretion of IL-1β by blocking the process of conversion of pro-interleukin 1β to mature interleukin 1β from caspase-1 [109,110,111,112].

Moreover, findings from a recent pilot study suggest that aspirin discontinuation may be feasible in patients with acute coronary syndrome undergoing PCI with DES implantation, with colchicine administered in combination with single antiplatelet therapy (ticagrelor or prasugrel). At 3-month follow-up, colchicine therapy was associated with a reduction in high platelet reactivity, defined as >208 P2Y12 reaction units (PRU), as well as a decrease in residual systemic inflammation, defined by CRP levels ≥ 2 mg/L. The primary endpoint occurred in two patients (1.0%), comprising one definite and one probable case of stent thrombosis [113]. These findings may provide a rationale for exploring novel therapeutic strategies in patients with contraindications to aspirin, including those at high risk of bleeding or with aspirin hypersensitivity. In such populations, the combination of single-antiplatelet therapy with targeted anti-cytokine treatment may represent a potential alternative therapeutic approach. Furthermore, these observations may provide new insights into the role of anti-cytokine therapies in the prevention and management of recurrent in-stent restenosis.

Interestingly, observational cohort studies have identified an elevated neutrophil-to-lymphocyte ratio (NLR) as an independent prognostic biomarker in ASCVD. Moreover, normalization of the NLR following anti-cytokine therapy has been associated with therapeutic responsiveness, suggesting that changes in this inflammatory index may serve as a potential biomarker of treatment efficacy [47]. Hence, the NLR is an inflammatory biomarker associated with MACE but not a measure of macrophage activity, thus the NLR has a lesser association with CAC whereas the RDW is associated with inflammation, insulin resistance, macrophage iron retention (increased ferritin levels) and increased polarization of the macrophages from M2 (inducing inflammation resolution) to M1 (triggering inflammation).

Therefore, the RDW has a greater association with CAC than NLR. In this regard, these findings suggest the hypothesis that a high RDW could be a promising target for anti-cytokine therapy, as well as that RDW normalization could be a potential marker of therapy responsiveness. Notwithstanding, the RDW is currently not validated as a treatment target, and more clinical trials are needed to explore this indication. Furthermore, given that high RDW is associated with high ferritin levels, especially in diabetes, some studies suggest the hypothesis that chelation therapy can reduce the risk of developing MACE, especially in patients with higher levels of insulin resistance, probably due to reducing the iron levels, and thus avoiding the polarization of the macrophages from M2 to M1. Hence, these findings suggest the hypothesis that diabetic patients with high RDW could be a potential target for chelation therapy. Nevertheless, further research is required to confirm whether reducing intracellular iron levels in macrophages with chelation therapy modulates the macrophages’ immune inflammatory activity by decreasing or blocking the release of proinflammatory cytokines and improves the prognosis in ASCVD either by reducing the risk of adverse cardiovascular events or the progression of coronary calcification.

## 10. RDW Clinical Perspective and Limitations

**Figure 3:**
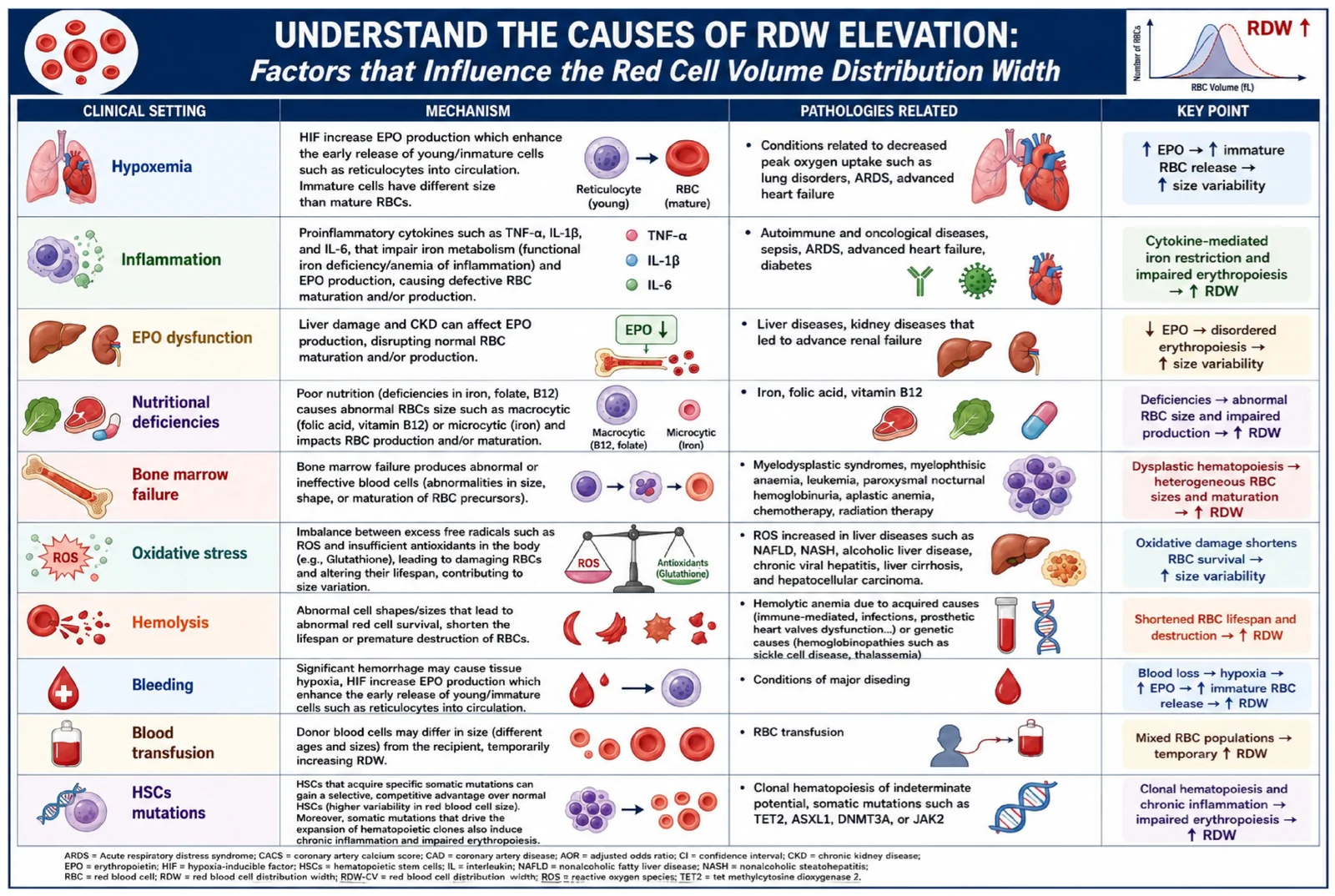
Causes of RDW elevation. The arrow ↑ means increase and the arrow → lead to.

**Figure 4:**
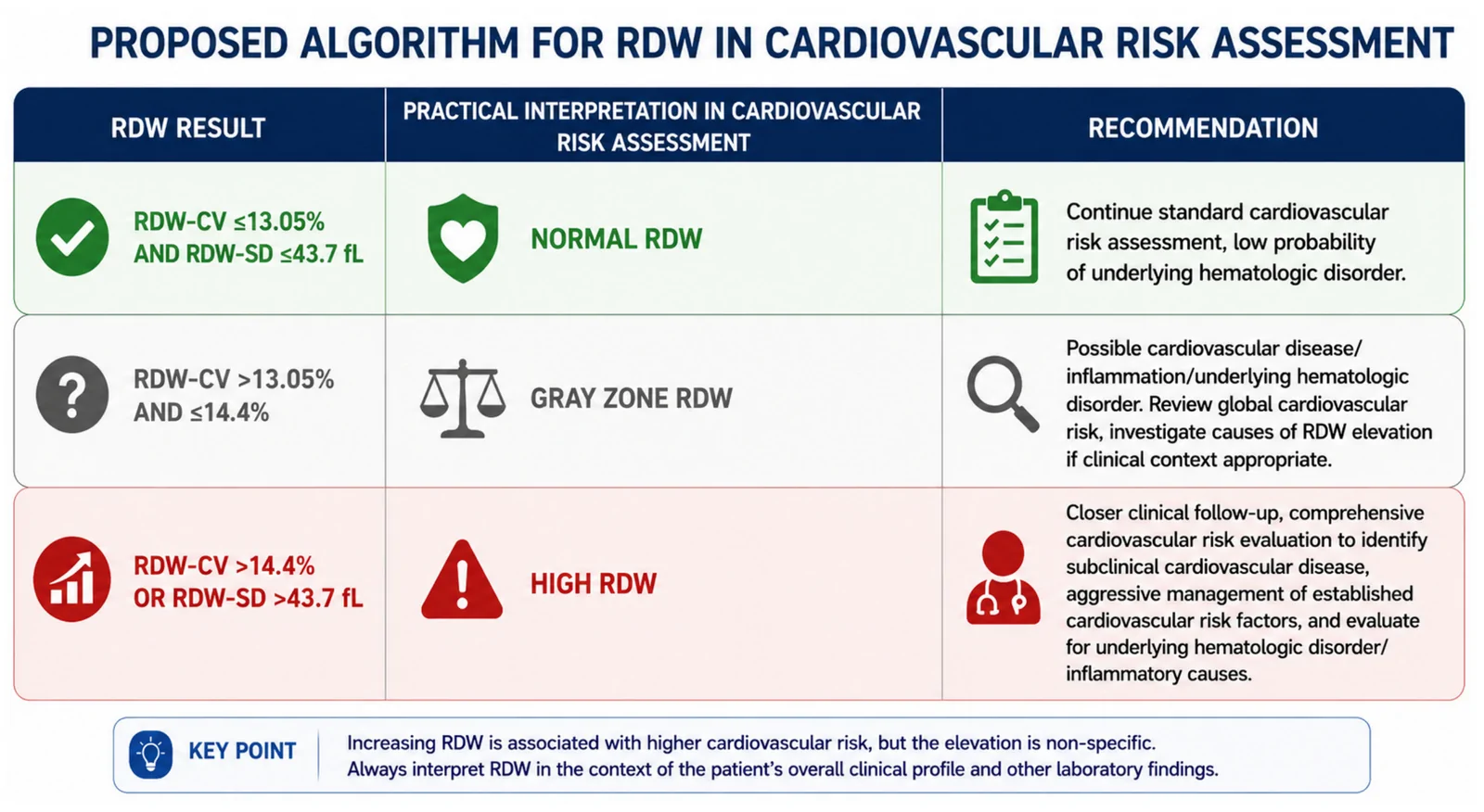
Proposed algorithm for RDW in cardiovascular risk assessment.

RDW in combination with mean corpuscular volume (MCV) has been used for the classification of anemias [87]. This is because RDW often rises earlier than other complete blood count indicators in nutritional deficiencies, especially in iron deficiency [87,88]. Thus, RDW may represent a sensitive hematological marker of iron deficiency, with a reported sensitivity of approximately 94%. Importantly, an RDW value within the normal reference range may help exclude iron deficiency in clinical settings where serum ferritin does not reliably reflect body iron stores because of inflammation, as commonly observed in patients with advanced HF [89]. Large clinical trials support the involvement of the inflammatory pathway mediated by the immune system in the development of ASCVD, which has led to high systemic inflammation as a novel cardiovascular risk factor and a target for anti-cytokine therapy [47]. Many studies have shown the association of high RDW with systemic inflammation, as well as its prognostic value in ASCVD, HF, and AF [20,87,88,89]. Some studies suggest that a reduction in the RDW is a potential predictor of iron replacement responsiveness in heart failure, and persistence of high RDW in AF after catheter ablation is associated with AF recurrence [87,89]. Therefore, a high RDW is a highly sensitive marker of inflammation that can be used to improve cardiovascular risk assessment in patients with established ASCVD as a prognostic marker, but it is not specific because other non-cardiovascular inflammatory causes and underlying blood disorders can sometimes raise RDW [37,40,87,88,89,90,91,92,102,114,115]. Therefore, all causes of high RDW must be considered to interpret whether the RDW elevation is due to inflammation, ineffective erythropoiesis, or both (Figure 3). Of note, based on the current evidence, an RDW-CV > 13.05% and ≤14.4% may be associated with at least mild CAC on computed tomography (CAC score > 0), as well as at least mild aortic valve calcification on multislice computed tomography (Table 1). An RDW-SD >43.7 fL is associated with a higher coronary artery calcium (CAC) score on computed tomography (CAC score > 100) [17,18], whereas an RDW-CV >14.4% is associated with an increased risk of adverse cardiovascular events [20,116]. Therefore, from a practical perspective, as proposed in the algorithm (Figure 4), patients with an RDW-CV > 14.4% or RDW-SD > 43.7 fL should be considered at increased cardiovascular risk and undergo comprehensive cardiovascular risk assessment, given the association of these values with adverse cardiovascular events, as well as the investigation of other causes of high RDW, especially underlying hematological disorders and other causes of inflammation (Figure 3). Moreover, the most commonly underlying blood disorders associated with CV RDW > 14.4% are iron deficiency (absolute or functional due to impaired iron mobilization from stores caused by inflammation), accelerated red blood cell turnover (acute blood loss recovery OR hemolytic anemia), ineffective bone marrow production (myelodysplastic syndromes, among others), and older adults who also have unexplained cytopenias may have CHIP and an increased risk of progression to myeloid disorders [87,88,89,114]. Notwithstanding, more clinical trials are required for standardizing cut-off values and validating them for diagnostic and therapeutic applications.

## 11. Conclusions

Considerable evidence supports the role of inflammation as an important factor that precedes and promotes vascular calcification. In this regard, extensive literature has shown that RDW is a prognostic marker in cardiovascular disease and is associated with inflammation. Although RDW is not a specific marker of cardiovascular disease and should not be interpreted as a direct mediator of vascular calcification, elevated RDW may serve as an integrative indicator of underlying inflammatory and hematopoietic disturbances that contribute to atherosclerotic disease. In particular, the proposed pathway linking IL-6-mediated hepcidin activation, macrophage iron retention, proinflammatory macrophage polarization, and osteogenic differentiation of vascular smooth muscle cells provides a biologically plausible mechanism connecting RDW with CAC. Observational evidence from eight cohort studies including 16,003 participants suggests that higher RDW-SD is associated with a greater burden of coronary calcification, whereas higher RDW-CV is associated with an increased risk of adverse cardiovascular events. From a practical perspective, high RDW values (RDW-CV > 14.4% or RDW-SD > 43.7 fL) have been associated with increased cardiovascular risk and adverse outcomes, including adverse cardiovascular events and all-cause mortality, and may justify closer clinical follow-up and more comprehensive cardiovascular risk assessment, as well as evaluation for underlying hematologic disorders and inflammatory causes. The hypothesis that RDW could identify patients who might benefit from anti-cytokine treatment or iron-modulating therapies remains unproven. More clinical trials are needed to validate the high RDW as a target for anti-cytokine and chelation therapy. Therefore, the novelty of this study is to propose that RDW is a useful marker of inflammation and a risk marker of CAC but has not yet been validated as a treatment target.

## Figures and Tables

**Table 1:** Studies related to red cell volume distribution width and its association with coronary artery calcification.

Author	RDW Cut-off Value	Study Method and Setting	Results and Conclusion
Jian et al. 2024 [17]	43.7 fL	Patients who underwent CT and subsequent invasive coronary angiography (*n* = 4796). Type of automated blood cell counter not reported.	Logistic regression analysis, each 1-SD increase in RDW-SD was associated with higher CACS > 400 (OR per 1-SD increase: 1.27, 95% CI: 1.20–1.33; *p* < 0.001) and after adjustment of other potential confounders including HTN, DM, DLP, smoker status, eGFR, homocysteine, triglycerides, RBC, BMI (adjusted OR per 1-SD increase: 1.11, 95% CI: 1.05–1.18; *p* < 0.001)
Jin et al. 2023 [18]	13.1% 44.2 fL	Hospitalized patients (*n* = 1257) who received a CT. automated blood cell counter (HmX AL Hematology Analyzer, Beckman Coulter, CA, USA)	High calcification group the RDW-SD and RDW-CV were significantly higher, than those in the low calcification group (*p* < 0.05). RDW-SD had a significant predictive value for severe calcification CACS > 400 (AOR = 2.571, 95% CI = 1.296–5.101; *p* < 0.01). adjustments were made for confounders (smoking, drinking, gender, age, DM, HTN, BMI, RBC, phosphorus, uric acid, calcium, CRP, eGFR)
Gürel et al. 2015 [21]	13.05%	Consecutive patients (*n* = 527) with a low to intermediate risk for CAD, underwent CT. automated blood cell counter (Beckman Coulter HmX AL Haematology Analyzer, Brea, CA, USA)	ROC analysis, a RDW value of 13.05% was identified as the best cut-off for predicting CACS > 100 (AUC = 0.706). CACS was positively correlated with patient age, HTN, DM, FRS, and it was inversely correlated with the ejection fraction, multivariate analysis showed that RDW (β = 0.156, *p* = 0.001), was independent predictor of the CACS
Oleksiak et al. 2016 [20]	14%	Consecutive patients (*n* = 269) with significant stable CAD. The primary endpoint was a composite of death or nonfatal myocardial infarction. Median post-discharge follow up was 43.2 months. automated complete blood Roche Cobas Analyzer	ROC analysis the best cut-off value for RDW for prediction of the primary event was 14% (AUC = 0.69; 95%CI:0.63–0.75; *p* = 0.0002) and for CACS was 603 (AUC = 0.66; 95%CI:0.60–0.72; *p* = 0.001).
Pan et al. 2022 [19]	46.8 fL	CT was performed and RDW was measured in subjects (*n* = 5772) from the Swedish CArdioPulmonary bioImage Study (SCAPIS). Sysmex XN-10 counter	CACS (>100) increased significantly with RDW: OR = 1.63, 95% CI: 1.29–2.05, *p* < 0.001. adjustments were made for confounders (age, sex, BMI, CRP, DLP, HTN, DM, eGFR, current smoking, anemia)
Roumeliotis et al. 2023 [22]	14.4%	CKD patients in pre-dialysis and dialysis (*n* = 158). Blood parameters that correlated with vascular calcification. automatic hematology analyzer Sysmex XE-5000 (Sysmex Corporation, Kobe, Japan)	multiple regression analysis showed RDW as independent variable. Multiple regression analysis showed that only circulating dp-ucMGP (a marker of vascular calcification in CKD) was an independent predictor of RDW (β = 0.001, *p* = 0.001), adjustments were made for confounders (albumin, CRP, UACR, calcium, phosphorus, cardiac rhythm, central diastolic blood pressure, warfarin, EPO)
den Harder et al. 2018 [23]	13.56%	Patients with suspected or known CAD who underwent CT CAC scoring and standard hematology analysis (*n* = 1504). automated blood cell counter Cell-Dyn Sapphire blood cell analyser (Abbott Diagnostics, Lake Forest, CA, USA)	RDW (β = 0.20 [0.05–0.36], *p* = 0.007), was positively associated with the CAC score after adjustment for age, sex, BMI, DM, eGFR, and HDL.
Zeng et al. 2024 [24]	44.66 fL 13.15%	Consecutive hospitalized patients (*n* = 1720) aortic valve calcification were assessed using MSCT. automated blood cell counter (HmX AL Blood Analyzer, Beckman Coulter, Brea, CA, USA)	RDW predicted aortic valve calcification. RDW–SD (AUC = 0.594, *p* < 0.001) and RDW–CV (AUC = 0.579, *p* < 0.001).

AOR = adjusted odds ratio; AUC = area under the curve; BMI = body mass index; CACS = coronary artery calcium score; CAD = coronary artery disease; CI = confidence interval; CKD = chronic kidney disease; CRP = C-reactive protein; CT = computed tomography; DM = diabetes; DLP = dyslipidemia; dp-ucMGP = dephosphorylated-uncarboxylated Matrix Gla-protein; eGFR = estimated glomerular filtration rate; EPO = Erythropoietin; FRS = framingham risk score; HDL = high-density lipoprotein; HTN = hypertension; RBC = red blood cell; RDW = red blood cell distribution width; RDW-CV = red blood cell distribution width coefficient of variation; RDW-SD = red blood cell distribution width standard deviation; OR = odds ratio; SD = standard deviation; ROC = receiver operating characteristic; UACR = urinary albumin–creatinine ratio.

## Data Availability

Any further data and material can be received through contacting the corresponding author.
